# Potential Implantable Nanofibrous Biomaterials Combined with Stem Cells for Subchondral Bone Regeneration

**DOI:** 10.3390/ma13143087

**Published:** 2020-07-10

**Authors:** Rana Smaida, Luc Pijnenburg, Silvia Irusta, Erico Himawan, Gracia Mendoza, Ezeddine Harmouch, Ysia Idoux-Gillet, Sabine Kuchler-Bopp, Nadia Benkirane-Jessel, Guoqiang Hua

**Affiliations:** 1 French National Institute of Health and Medical Research (INSERM), UMR 1260, Regenerative Nanomedicine (RNM), FMTS, 11 rue Humann, 67000 Strasbourg, France; rana.smaida@etu.unistra.fr (R.S.); luc.pij@gmail.com (L.P.); ezeddine.harmouch@etu.unistra.fr (E.H.); yidouxgillet@unistra.fr (Y.I.-G.); kuchler@unistra.fr (S.K.-B.); 2 Faculté de Chirurgie Dentaire de Strasbourg, Université de Strasbourg, 8 rue Sainte-Elisabeth, 67000 Strasbourg, France; 3 Department of Chemical Engineering, Aragon Institute of Nanoscience (INA), University of Zaragoza, Campus Río Ebro-Edificio I + D, C/Poeta Mariano Esquillor S/N, 50018 Zaragoza, Spain; sirusta@unizar.es (S.I.); erico.himawan@gmail.com (E.H.); 4 Aragon Health Research Institute (IIS Aragon), 50009 Zaragoza, Spain; gmendoza@iisaragon.es; 5 Networking Research Center on Bioengineering, Biomaterials and Nanomedicine, CIBER-BBN, 28029 Madrid, Spain

**Keywords:** biomaterial, bone engineering, cartilage, sodium hyaluronate, hydroxyapatite, osteochondral defect, polycaprolactone, regeneration, scaffold, stem cells, subchondral bone

## Abstract

The treatment of osteochondral defects remains a challenge. Four scaffolds were produced using Food and Drug Administration (FDA)-approved polymers to investigate their therapeutic potential for the regeneration of the osteochondral unit. Polycaprolactone (PCL) and poly(vinyl-pyrrolidone) (PVP) scaffolds were made by electrohydrodynamic techniques. Hydroxyapatite (HAp) and/or sodium hyaluronate (HA) can be then loaded to PCL nanofibers and/or PVP particles. The purpose of adding hydroxyapatite and sodium hyaluronate into PCL/PVP scaffolds is to increase the regenerative ability for subchondral bone and joint cartilage, respectively. Human bone marrow-derived mesenchymal stem cells (hBM-MSCs) were seeded on these biomaterials. The biocompatibility of these biomaterials in vitro and in vivo, as well as their potential to support MSC differentiation under specific chondrogenic or osteogenic conditions, were evaluated. We show here that hBM-MSCs could proliferate and differentiate both in vitro and in vivo on these biomaterials. In addition, the PCL-HAp could effectively increase the mineralization and induce the differentiation of MSCs into osteoblasts in an osteogenic condition. These results indicate that PCL-HAp biomaterials combined with MSCs could be a beneficial candidate for subchondral bone regeneration.

## 1. Introduction

Regenerative medicine is a multidisciplinary domain that has emerged as a result of new advances in stem cells and biomaterials to achieve the goal of cell and organ replacement to regain function resulting from degeneration, aging, trauma, or other diseases. Osteoarthritis (OA) is one of the most widespread chronic diseases in adults around the world. About 20% of adult people will be affected by OA in industrialized countries by 2030. This disease can occur as a result of ageing, obesity, or as a consequence of joint traumatic injury. The knee joint, the most affected by OA, represents a major cause of disability, but also cardiovascular mortality due to OA-induced sedentary lifestyle [1]. A joint mainly consists of bone (periarticular and subchondral), cartilage, muscles, the synovial stratum, and an articular capsule. OA is characterized by a progressive destruction of cartilage but also affects subchondral bone, synovial membrane, periarticular soft tissues, and causes high levels of pain in joints, as well as stiffness, loss of function, effusion, bone enlargement, and misalignment, impacting the patients’ quality of life [2].

Current therapies to treat early stage of OA aims at stopping joint degradation, instead of just treating symptoms [3]. Mesenchymal stem cells (MSCs) isolated from bone marrow or adipose tissue secrete a large amount of growth factors and can differentiate into other cell types, such as osteoblasts and chondrocytes [4], thus possessing good regenerative properties [5]. By intra-articular injection, these cells are reported to influence the course of the disease and to prevent cartilage degradation through their paracrine potential and their interactions with neighboring cells, consequently improving the knee function and structure [6]. However, although MSC-based treatment seemed promising, failures in joint regeneration are mainly related to the fact that the subchondral bone is not able to support the regeneration of articular cartilage because it is known that most chondral lesions caused by degradation of joint cartilage affect the subchondral bone.

Recently, several therapeutical innovative strategy combining the biomaterials-based scaffolds and MSCs for osteoarticular regeneration has been reported [7,8,9,10,11,12,13,14]. Many biomaterials, such as bioceramics, biopolymers, metal, and composites, have been identified as appropriate for bone tissue engineering and MSC seedling. The nanofibrous scaffolds can be prepared by many methods, such as phase separation, self-assembly, and electrospinning [15]. The electrospinning process, which is simple, flexible and cost-effective, has been intensively studied due to its ability to produce nanofibers with morphological structures close to those in a natural extracellular matrix (ECM) and suitable for a range of cellular functions [16]. Electrospun nanofibers have an incredibly high functional surface area, which enables the scaffolds to imitate the ECM collagen. They have great interconnectivity of pore displaying a superposition of thin nanofiber layers. Interactions amongst different nanofiber layers arise spontaneously throughout the electrospinning process, resulting in variable pore size distribution, thus mimicking the micro- to nanoscale topography of the extracellular matrix thereby facilitating cell adhesion, cell development, and vascularization [17,18]. Such specific properties of nanofiber-based implants are attractive features for innovative applications. The electrodynamic technique also allows the production of polymer nanoparticles that can be used for the encapsulation of small molecules, proteins, theranostic elements, nucleotides and other therapeutics [19]. Electrosprayed reservoirs can be synthesized with desired size, shape, proper loading of drug molecules into the spear, and bioactive ingredients immobilization [20].

The essential property for biomaterial-based scaffolds used in bone tissue engineering is biocompatibility, however, the three-dimensional (3D) microenvironment inside the scaffolds should support cell adhesion, proliferation, differentiation and extracellular matrix (ECM) formation. In addition, the scaffolds should also be bioactive (osteoconductive, enabling bone cells to grow and/or osteoinductive, triggering new bone formation) and biodegradable at the rate of new bone formation. It is important to note that the microenvironment dictated by the physical and chemical properties of the scaffold plays a key role in cell function and eventual tissue regeneration. Soft flexible membrane-based scaffolds could be introduced into the defect through less intrusive methods and promote bone development by inducing osteoconductivity and creating a barrier to soft tissue growth [21].

Hydroxyapatite (HAp) has gained interest as a scaffold material for bone regeneration thanks to its exemplary biocompatibility with hard tissue, high osteoconductivity and bioactivity, without neither antigenic nor cytotoxic effect. HAp increases the bioactivity of the scaffold by providing a source of calcium and phosphate ions that can be used by osteogenic cells to form bone [22]. Hyaluronic acid (HA) is an essential element of synovial fluid that preserves joint cartilage by lubricating and resisting shocks [23]; however, unfortunately, the intrinsically regenerative and damage-repair ability of human hyaline cartilage is restricted [24]. HA normally retains a constant concentration and adequate viscosity in articulations, but, when OA arises, the concentration of HA decreases, which exacerbates knee cartilage damage. This is the reason why OA patients may improve with HA supplementation [23,25].

In this context, the objective of this study was to investigate the potential bone and/or cartilage regenerative properties of a scaffold made of two FDA-approved polymers, polycaprolactone (PCL) and poly(vinyl-pyrrolidone) (PVP), using electrohydrodynamic techniques. Then, hydroxyapatite (HAp) could be loaded to PCL fibers and PVP particles could be charged by sodium hyaluronate (HA). Both materials were obtained at the same time by electrospinning and electrospraying, respectively. To analyze the effect of the different types of the scaffold, four biomaterials were prepared: PCL fiber with PVP particles (hereafter called PCL), HAp loaded PCL fibers decorated with PVP particles (hereafter called PCL-HAp), PCL fibers with HA loaded PVP particles (hereafter called PCL-HA), and HAp loaded PCL fibers decorated with HA loaded PVP particles (hereafter called PCL-HAp-HA). All the prepared biomaterials were characterized by Scanning Electron Microscopy (SEM), and the content of bioactive molecules was measured. We evaluated, both in vitro and in vivo, the biocompatibilities of these biomaterials, the proliferation, and differentiation of MSCs seeded on these biomaterials.

## 2. Materials and Methods

### 2.1. Materials

Polycaprolactone (PCL, MW: 80,000 g/mol), hydroxyapatite nanoparticles (HAp, average diameter; 200 nm), dimethylformamide (DMF), dichloromethane (DCM), polyvinylpyrrolidone (PVP. MW: 55,000), and ethanol 99% were from Sigma Aldrich (Darmstadt, Germany). Sodium hyaluronate (HA) (HA100K MW: 100–150 kDa) was provided by Lifecore Biomedical (Chaska, MN USA).

### 2.2. Preparation of Scaffold

Four different types of scaffolds (PCL, PCL-HAp, PCL-HA, PCL-HAp-HA) were used, Table 1. Scaffolds were prepared by electrospinning process using an Yflow 2.2. D-500 electrospinner (Coaxial Electrospinning Machines/R&D Microencapsulation, Valencia, Spain). To obtain PCL scaffolds, PCL pellets were dissolved at 10% w/w (PCL/solvents) in dichloromethyl/dimethylformamide (DCM/DMF) (1:1); the solution was stirred overnight at room temperature. For electrospinning of PCL-HAp membrane scaffold, PCL polymer solutions (10% *w*/*v*) with hydroxyapatite powder was prepared under constant stirring at room temperature. The solvent used was a mixture of DCM and DMF (3:1). Hydroxyapatite nanoparticles were thoroughly mixed in the solvent and the PCL pellet mass required to reach 10% *w*/*v* polymer concentration was added gradually. For electrospraying, PVP solution (3% *w*/*v*) was prepared by dissolving the polymer powder in a mixture of ethanol and water as solvent (EtOH:H_2_O = 8.5:1.5). To incorporate the sodium hyaluronate into PVP particles, HA salt was firstly dissolved in the solvent and the resulting solution was used to dissolve PVP. The mixture solution was stirred at room temperature for 2 h prior to electrospraying. All solutions were loaded into plastic syringes. To obtain PCL-HAp fibers, PCL-HAp suspension was fed through the needle (1 mL/h). PCL scaffolds decorated with PVP particles, both loaded and unloaded, produced following the protocol described in previous studies [26]. Two needles were used to obtain the PCL fibers (needle 1) and to electrospray the HA loaded PVP particles (needle 2). Through needle 1, PCL or PCL-HAp solution was fed at 1 mL/h, while, through needle 2, PVP or PVP-HA solution was fed at 0.3 mL/h. Both needles were connected to the positive power supply at a voltage of 12.25 kV. The tips of the needles were fixed 14 cm above a rotating collection drum at 100 rpm. The negative voltage power supply (−3.3 kV) was connected to the collector.

### 2.3. Physicochemical Characterization

Morphology of PVP particles and electrospun scaffolds was analyzed using a SEM microscope (Field Emission Scanning Electron Microscope CSEM-FEG INSPECT 50, FEI, Hillsboro, OR, USA). Samples were sputtered with gold and platinum metal prior observation. The size distribution statistics were obtained by measuring at least 100 fibers or particles in different images using ImageJ software (Version 1.48f, NIH, Bethesda, MA, USA). Thermogravimetric analysis (TGA) was performed to evaluate the HAp content of the scaffolds using a METTLER TOLEDO (TGA/SDTA 851, Mettler Toledo, Barcelona, Spain). Samples were analyzed in a Waters 2695 chromatograph equipped with a Waters 2295 PDA detector to measure HA scaffolds load. The elution was performed at 30 °C through a Fortis Bio C4 column using water (0.1%*w*/*v* NH_4_Cl) as eluent at a flowrate of 0.3 mL/min.

### 2.4. In Vitro Study

Samples were cut using a biopsy punch for the different studies, with a diameter of 12 mm for in vitro studies and 6 mm diameter for in vivo subcutaneous implantations. Before any in vitro or in vivo use, we proceeded to sterilize the scaffolds with 70% ethanol for 10 min, and each side of the scaffold was exposed for 30 min to ultraviolet (UV) light.

### 2.5. Cell Seeding

In vitro cell culture studies were conducted using human mesenchymal stem cells, derived from bone marrow (hBM-MSCs, PromoCell, Heidelberg, Germany) ranging from passage 3 to 6. We performed the expansion of the MSCs in T75 tissue culture flasks (TC Flask, Sarstedt, Germany) using Mesenchymal Stem Cell Growth Medium MSC2 (PromoCell, Heidelberg, Germany), without antibiotics or antifungals. Once at confluence, the cells were collected using the enzyme Trypsin (Lonza, Walkersville, MD, USA). 20 × 10^3^ cells/cm^2^ were seeded on the sterilized electrospun scaffolds. The 4 types of scaffolds were then cultured for 3 days in proliferation medium MSC2 and then in osteogenic or chondrogenic differentiation basal medium. Only the differentiation media contained antibiotics (Penicillin and Streptomycin 100 μg/mL). The medium was renewed three times a week during the 21 days of culture.

### 2.6. Biocompatibility and Proliferation

The 4 types of scaffolds were rinsed with phosphate buffered saline (PBS) and then incubated 10% AlamarBlue^®^ (Thermo Fisher Scientific, Eugene, OR, USA)/Dulbecco’s Modified Eagle’s Medium phenol red-free culture medium (Lonza, Biowhittaker, Belgium) solution, in a humid atmosphere at 37 °C and 5% CO_2_. After 4 h incubation, 200 µL of medium from each well was transferred to 96-well plates, and the optical density of that supernatant was then measured at 2 wavelengths, 570 nm (reduced product absorbance) and 595 nm (oxidized product absorbance), by spectrophotometry (Thermo Fisher Scientific, Multiskan FC Microplate Photometer, Vantaa, Finland). The evaluation was conducted after 3 days, 7 days, 14 days, and 21 days of culture in an osteogenic or chondrogenic culture medium. The percentage of reduction was then calculated and correlated to the metabolic activity of the cells (n = 3). The statistical analysis was performed by a t-test (student test for matched data) on BiostaTGV.

### 2.7. Real-Time Quantitative PCR (RT-qPCR)

RT-qPCR was performed to evaluate the messenger Ribonucleic Acid (mRNA) expression of alkaline phosphatase (ALP), Bone sialoprotein II (BSPII), Runt-related transcription factor 2 (RUNX2), and Collagen type II(COLII). Total RNA was extracted using TRIzol reagent (Invitrogen) as instructed by the manufacturer. RNA concentrations and purity were measured using the NanoDrop ND-1000 spectrophotometer (NanoDrop Technologies, Rockland, DE, USA). Reverse transcription was conducted with the iScript^®^ reverse Transcription Supermix (Bio-Rad, Marnes-la-Coquette, France). The real-time PCR reaction was then performed using the iTaq^®^ Universal SYBR^®^ green super mix (Bio-Rad) and the CFX cycler system (Bio-Rad) with the following cycle conditions: an initial denaturation step of 95 °C was performed for 2 min, followed by 39 cycles of denaturation at 95 °C for 5 s, annealing at 60 °C during 30 s, and extension at 65 °C for 5 s. The experiment was performed three times.

To quantify the expression of RNA, qPCR was performed on the complementary Deoxyribonucleic Acid (cDNA) samples. PCR amplification and analysis were achieved using the CFX Connect™ Real-Time PCR Detection System (Bio-Rad, Miltry-Mory, France). Amplification reactions were conducted using iTaq Universal SYBR Green Supermix (Bio-Rad, Miltry-Mory, France). Beta-actin was used as the endogenous RNA control (housekeeping gene) in the samples. Primer sequences were synthesized by Life Technologies (Saint-Aubin, France). The specificity of the reaction was controlled using melting curve analysis. The expression level was calculated using the comparative Ct method (2^−ΔΔCt^) after normalization to Beta-actin. All PCR assays were performed in triplicate, and the results are represented by the mean values. All primers sequences are listed in Table 2.

### 2.8. Histological Mineralization Analysis

All samples were fixed with 4% paraformaldehyde (PFA), rinsed with distilled water once, then stained with a 1% Alizarin Red S (AR) solution (pH 4.2 Sigma-Aldrich, Saint-Quentin Fallavier, France) for 20 min at room temperature. After the removal of AR solution, the samples were rinsed several times with distilled water. This staining was carried out on the scaffolds grown for 10 and 21 days in osteogenic or chondrogenic culture medium. An Alkaline Phosphatase Assay was performed, as well, on the hBM-MSCs grown for 7 days on the different scaffolds. Samples were first washed with PBS and then fixed with 10% formalin for 60 s. They were then washed with a washing buffer consisting of 0.05% Tween 20/Dulbecco’s PBS without Ca++/Mg++. Cells were stained using a BCIP (5-brom-4-chloro-3’-indolyphosphate p-toluidine salt)/NBT (nitro blue tetrazolium chloride) substrate solution (SigmaFast^TM^ BCIP/NBT; Sigma Aldrich) at room temperature in the dark for 10 min. After the removal of the BCIP/NBT solution, the samples were washed with the washing buffer and then with PBS.

### 2.9. Osteogenic and Chondrogenic Differentiation Analysis by Immunofluorescence

The scaffolds were first rinsed with phosphate buffered saline (PBS) 2 times, then fixed in paraformaldehyde (PFA) solution (4%) for 15 min. Samples were then incubated in a saturation/permeabilization solution (1% bovine serum albumin(BSA)/0.1% Triton-X, PBS) for 30 min followed by 3 PBS washes. Afterwards, the samples were stained for 2 h at room temperature with rabbit primary antibodies (1/200) for the detection of osteogenic differentiation markers: the bone sialoprotein BSPII molecule (Rabbit, Santa Cruz Biotechnology), the transcription factor RUNX2 (Rabbit, Sigma-Aldrich), and a chondrogenic differentiation marker: type II collagen (COLII, Rabbit, Sigma-Aldrich). After washing with PBS 3 times, the revelation was made by secondary anti-rabbit antibodies conjugated to Alexa 488 (1/200) (Molecular Probes, Invitrogen). Additional labelling of the nuclei with 4′,6-diamidino-2-phenylindole (DAPI) (200 nM, Sigma-Aldrich, Saint-Quentin Fallavier, France) was performed. The samples were mounted on slides (Dako^®^, Courtaboeuf, France) and observed with an epifluorescence microscope (Leica DM 4000B, Nanterre, France).

### 2.10. In Vivo Study

The experimental protocol fulfilled the authorization of the “Ministère de l’Enseignement Supérieur et de la Recherche” under the agreement number 01716.02. The Ethics Committee of Strasbourg named “Comité Régional d’Ethique en Matière d’Expérimentation Animale de Strasbourg (CREMEAS)” specifically approved this study.

### 2.11. Subcutaneous Implantation in Mice to Evaluate Biocompatibility and Mineralization

Each mouse was given 4 sample implants in a single operation (one type of membrane per sample). After asepsis measurements, 4 dorsal skin incisions were made, and the sterilized scaffolds (6 mm diameter discs, 50 μm thick) were inserted at the distant site from the incision. The skin was then sutured with VicrylTM 5.0 resorbable sutures. All procedures were performed under general anaesthesia with ketamine (100 mg/kg, Virbac Santé Animale, Centravet, Nancy, France) and xylazine (10 mg/kg, 2%, Rompun, Bayer Healthcare, Animal Health Division, France) injected intraperitoneally. Ten-week-old ICR mice (CD-1 mouse, Charles River, L’Arbresle, France) (n = 8) were each implanted with the four types of scaffolds for 2 weeks (n = 4 mice) or 4 weeks (n = 4 mice). Nude mice (n = 8) (NIH-Foxn1nu, Charles River, L’Arbresle, France) aged 4 weeks each also benefited from implantation of the 4 types of membranes previously inoculated with 3000 hBM-MSCs for 2 weeks. After sacrificing the mice by intraperitoneal injection of sodium pentobarbital (140 mg/kg, EUTHASOL VET^®^ Solution injectable, DECHRA Veterinary Products SAS), the total dorsal skin flap was removed to relocate the implants. The scaffolds and adjacent tissues were collected and fixed in paraformaldehyde (4% in PBS) overnight at 4 °C. Histological sections of these samples were then cut (10 μm), after paraffin inclusion, using the microtome. Hematoxylin-Eosin (HE) and Alizarin Red S (AR) were performed for standard histological structures and detection of mineralization, respectively.

## 3. Results

### 3.1. Structure of the Different Scaffolds

**Figure 1:**
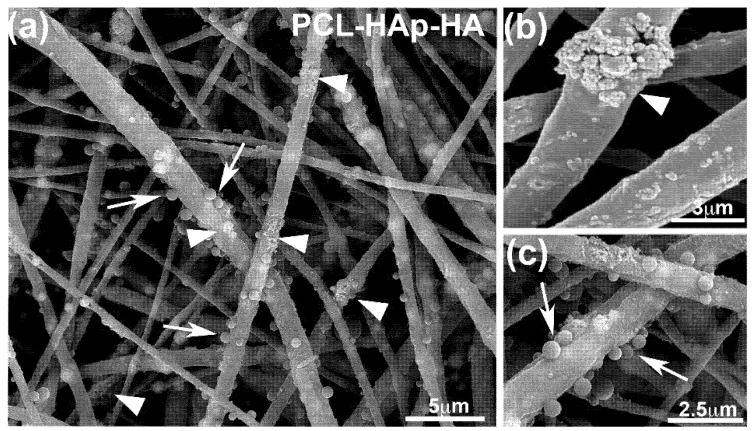
SEM images of polycaprolactone (PCL)-hydroxyapatite (Hap)-hyaluronic acid (HA) scaffold. Arrowheads point to HAp in (**a**,**b**) and arrows point to poly(vinyl-pyrrolidone) (PVP) particles in (**a**,**c**).

Scaffolds structure was analyzed by Scanning Electron Microscopy. PCL fibers were randomly oriented with an average diameter of 992 ± 318 nm (Figure 1). HAp particles can be observed inside the polymer fibers and also forming agglomerates on fibers surface (Figure 1a,b). HA-loaded PVP particles with average diameter of 436 ± 86 nm are attached along the fibers surface (Figure 1a,c). Using TGA for HAp and chromatography for HA, loading efficiencies were calculated as:

(1) $$LE=\frac{experimentally\;measured\;mass}{theoretical\;mass}\times 100.$$

For hydroxyapatite, a LE of 93.99 ± 0.82 was achieved, while, for HA, the LE was 94.98 ± 1.12%. That LE implies a HAp content in the scaffold PCL-HAp-HA of 35.33 ± 0.31 wt.%, while, for HA, the load was 0.14 ± 0.002 wt.%.

### 3.2. In Vitro Biocompatibility of the Scaffolds

**Figure 2:**
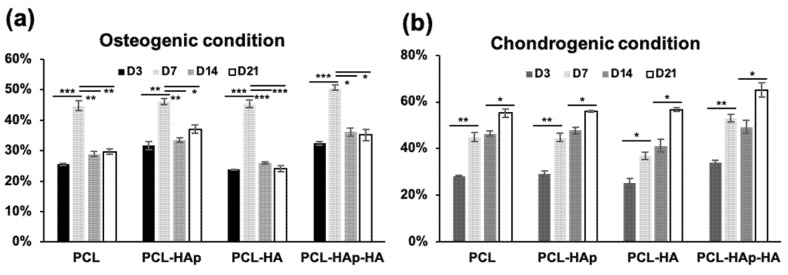
Metabolic activity of human bone marrow-derived mesenchymal stem cells (hBM-MSCs) grown on the PCL, PCL-HAp, PCL-HA, and PCL-HAp-HA scaffolds, in an osteogenic medium (**a**) and a chondrogenic medium (**b**) (presented as the percent of reduction by AlamarBlue^®^). Data are represented as mean ± SEM of at least three independent experiments. The significance of the difference was calculated by student test, * *p* < 0.05; ** *p* < 0.1, and *** *p* < 0.01.

In order to evaluate the proliferation of human bone marrow-derived MSCs on PCL, PCL-HAp, PCL-HA, and PCL-HAp-HA nanofibrous scaffolds, MSCs were seeded on these four membranes and cultured in either osteogenic medium or chondrogenic medium for three weeks. The metabolic activities of MSCs were then assessed by AlamarBlue^®^ test. As shown in Figure 2a, the percent of AlamarBlue^®^ reduction significantly increased from D3 to D7, which indicated a good proliferation of MSCs in the first week. Interesting, this percent of reduction drastically reduced after 2 weeks’ (D14) or 3 weeks’ (D21) culture of MSCs, indicating that the metabolic activities were reduced. In great contrast, MSCs cultured in chondrogenic medium continued to proliferate, which has been revealed in Figure 2b by the continuous increase of the percent of reduction of AlamarBlue activity. However, the same profile was observed for all the four membranes tested in either osteogenic or chondrogenic condition, suggesting that adding HAp and/or HA did not affect the metabolic activity of MSCs. These results also suggested that the differentiation of MSCs could have occurred after one-week culture in osteogenic condition but not in chondrogenic condition.

**Figure 3:**
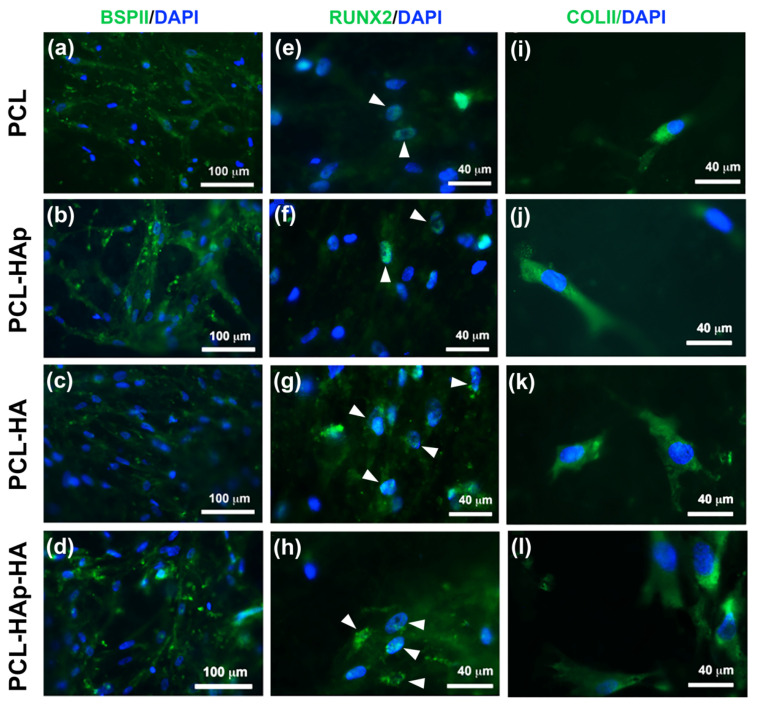
Indirect immunofluorescence of bone specific proteins BSPII (**a**–**d**) and RUNX2 (**e**–**h**) and chondrogenic protein Collagen type II (COLII) (**i)–(l**) on the hBM-MSCs seeded scaffolds: PCL, PCL-HAp, PCL-HA, and PCL-HAp-HA in an osteogenic medium (**a**–**h**) or chondrogenic medium (**i**–**l**) for 21 days. The white arrowheads indicate the nuclear staining (**e**–**h**). The nuclei are stained in blue (DAPI).

Thus, after 21-day’s culture in either osteogenic or chondrogenic medium, indirect immunofluorescence was performed with MSCs seeded on these four biomaterials. Bone-specific marker BSPII, as well as osteoblast differentiation specific marker RUNX2, were used in the osteogenic condition, and cartilage specific marker Collagen type II (COLII) was used in the chondrogenic condition. Both BSPII and RUNX2 staining were observed in cells under the osteogenic condition for all membranes (Figure 3a–h). Interestingly, the expression of both BSPII and RUNX2 proteins were stronger in the membrane containing HAp (PCL-HAp and PCL-HAp-HA) as compared to PCL and PCL-HA, indicating that HAp could indeed promote the osteogenic differentiation of MSCs. The COLII staining was also observed under chondrogenic condition for all the four membranes tested; however, no significant difference was observed (Figure 3i–l). These results demonstrated that MSCs could differentiate into specific cell types under specific condition on these biomaterials. It is worth noting that the presence of HAp could induce the osteogenic differentiation of MSCs.

**Figure 4:**
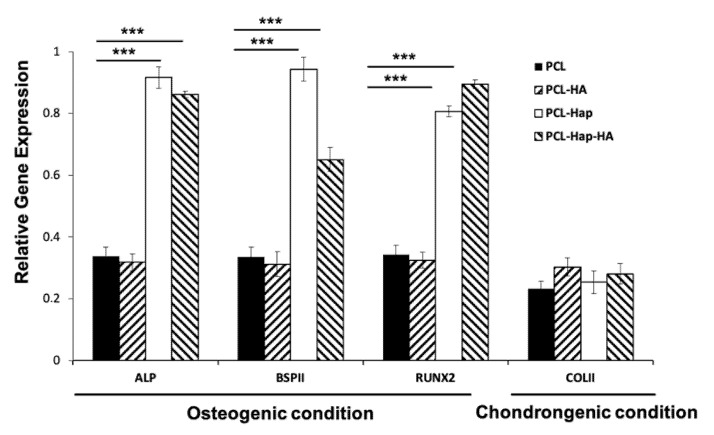
Relative gene expression of alkaline phosphatase (ALP), BSPII, RUNX2, and COLII genes in hBM-MSCs cultured for 7 days in osteogenic and chondrogenic conditions. *** *p* < 0.01 as compared to PCL.

Quantitative PCR analyses were further performed with cells cultivated on these four biomaterials under the osteogenic and chondrogenic conditions for 3 and 7 days. In agreement with the results obtained from immunofluorescence analyses, the expression of BSPII and RUNX2 genes, as well as that of the bone mineralisation marker Alkaline phosphatase (ALP), were significantly increased at both 3 days (see Supplementary Figure S1) and 7 days (Figure 4) on PCL-HAp and PCL-HAp-HA. However, the cartilage specific marker COLII was not significantly induced under the chondrogenic condition on PCL-HA and PCL-HAp-HA.

**Figure 5:**
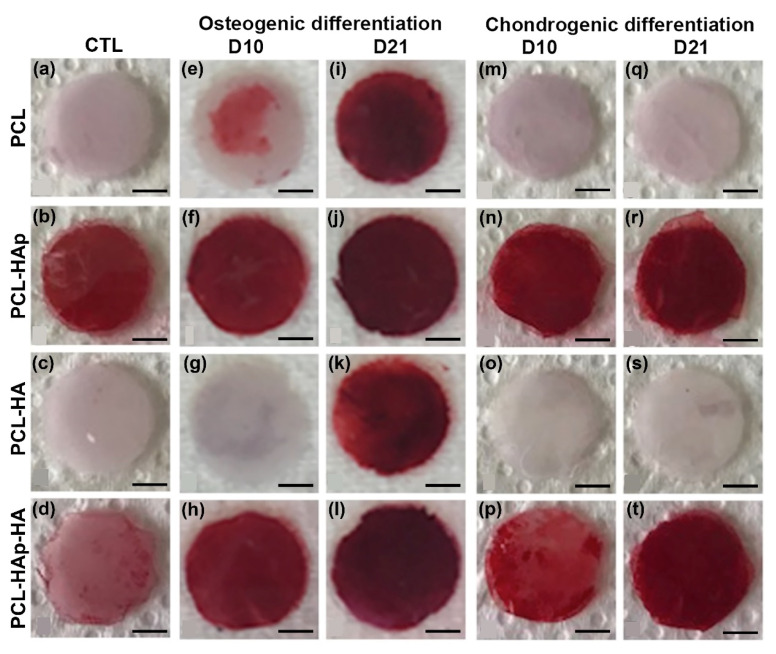
Staining with Alizarin Red S after 10 days (**e**–**h**), (**m**–**p**) and 21 days (**i**–**l**), (**q**–**t**) of culture of hBM-MSCs on the four types of scaffolds in an osteogenic medium (**e**–**l**) and a chondrogenic medium (**mt**). Control cell-free membranes were also stained (**a**–**d**). Bars represent 2 mm.

In order to investigate whether the human bone marrow-derived MSCs seeded on these four biomaterials could differentiate into osteoblasts in vitro, Alizarin Red S staining and Alkaline Phosphatase Activity Assay were carried out directly on all scaffolds to evaluate the degree of mineralization. Not surprisingly, the Alizarin Red S stain was observed on the PCL-HAp and PCL-HAp-HA control scaffold without cells, but not on PCL or PCL-HA scaffolds, due to the presence of HAp in the scaffolds (Figure 5b,d). This color became more intensified over the days of cell culture in PCL, PCL-Hap, and PCL-Hap-HA scaffolds in osteogenic medium (D10 and D21) but only appears after 3-weeks’ culture on PCL-HA scaffolds (Figure 5e–l). Importantly, weaker stain was observed in PCL and PCL-HA scaffolds as compared to those in PCL-HAp and PCL-HAp-HA scaffolds. The same results were observed with Alkaline Phosphatase activity assay (Supplementary Figure S2). As a negative control, no Alizarin Red S stain was observed on PCL and PCL-HA scaffolds in chondrogenic medium (Figure 5m,o,q,s). However, the Alizarin Red S stain was observed on the HAp containing scaffolds under chondrogenic condition, which, in addition, appeared to intensify with time (Figure 5n,p,r,t). These results demonstrated that these four biomaterials could indeed support the differentiation of MSCs into osteoblasts in an osteogenic environment. The presence of HAp could effectively stimulate the osteogenic differentiation, even under a chondrogenic condition.

Taken together, these results clearly showed a good biocompatibility of these four biomaterials which could promote both proliferation and differentiation of human bone marrow-derived MSCs in vitro.

### 3.3. In Vivo Biocompatibility of the Scaffolds

**Figure 6:**
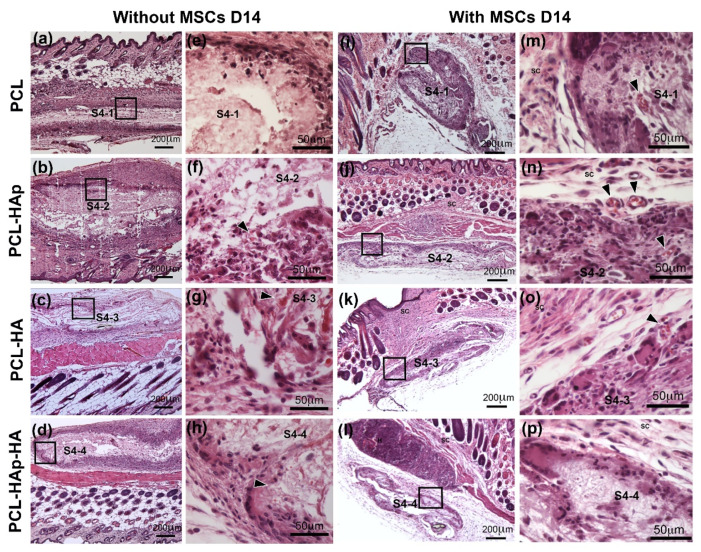
Haematoxylin-Eosin (HE) staining for PCL, PCL-HAp, PCL-HA, and PCL-HAp-HA scaffolds subcutaneously implanted for 2 weeks without cells in ICR mice (**a**–**h**) and with hBM-MSCs in nude mice (**i**–**p**). S4-1 in (**a**,**e**,**i**,**m**) for the PCL scaffold; S4-2 in (**b**,**f**,**j**,**n**) for the PCL-HAp scaffold; S4-3 in (**c**,**g**,**k**,**o**) for the PCL-HA scaffold; and S4-4 in (**d**,**h**,**l**,**p**) for the PCL-HAp-HA scaffold. Black arrowheads indicate vascularization. Black squares indicate the enlarged areas.

To determine the in vivo biocompatibility of these four scaffolds, these sterilized scaffolds with or without human bone marrow-derived MSCs were subcutaneously implanted in ICR mice (without hMSCs) (Figure 6a–h) or nude mice (with hMSCs) (Figure 6i–p). Two-week’s post-implantation, mice were sacrificed and transplanted membranes were taken out for histological analyses. HE staining revealed that, in neither mouse models, with or without hMSCs, no foreign body granulomas, no scarring incidents, nor signs of rejection were observed at the implantation site (see Supplementary Figure S3). Good integration of the scaffold into the subcutaneous scar tissue was also observed with the existence of peripheral vascularization predominantly involves the PCL scaffold (Figure 6f–h,m–o, black arrowheads). Thus, these results demonstrated a good biocompatibility of these four biomaterials in vivo.

### 3.4. HAp and hBM-MSCs Can Accelerate in Vivo Mineralization

**Figure 7:**
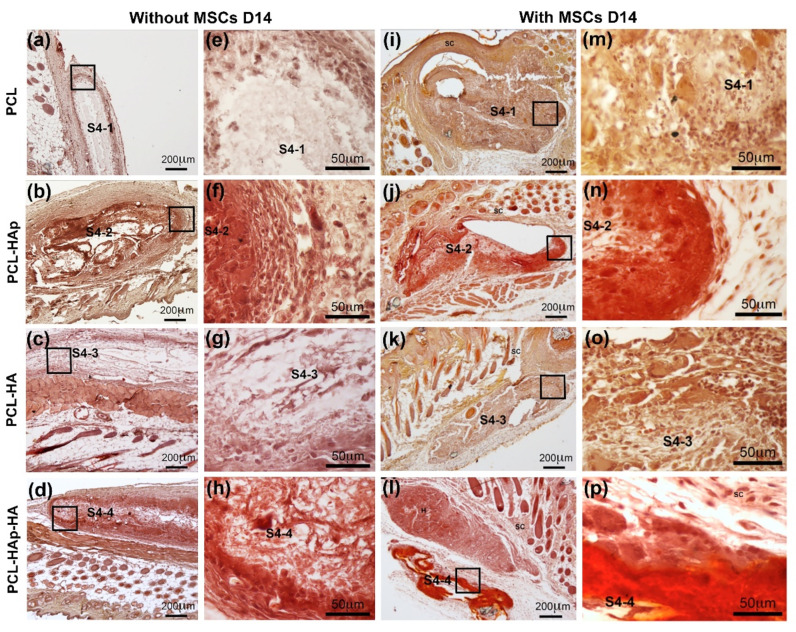
Histological staining with Alizarin Red S for PCL, PCL-HAp, PCL-HA, and PCL-HAp-HA scaffolds subcutaneously implanted without cells in ICR mice (**a**–**h**) and with hBM-MSCs in nude mice (**i**–**p**) for 2 weeks. (**a**,**e**,**i**,**m**) S4-1 for the PCL scaffold; (**b**,**f**,**j**,**n)** S4-2 for the PCL-HAp scaffold; (**c**,**g**,**k**,**o**) S4-3 for the PCL-HA scaffold; and (**d**,**h**,**l**,**p**) S4-4 for the PCL-HAp-HA scaffold. Black squares indicate the enlarged areas.

To further study the in vivo mineralization of these biomaterials and the osteogenic differentiation of the human bone marrow-derived MSCs on these biomaterials, sterilized scaffolds seeded with or without cells were subcutaneously implanted either in ICR mice (without hMSCs) or in Nude mice (with hMSCs). Two weeks’ post-implantation, in agreement with the results obtained from in vitro experiments (see Figure 5), a more intensified Alizarin Red S Stain was observed in biomaterials containing HAp (PCL-HAp and PCL-HAp-HA) (Figure 7b,d,f,h,j,l,n,p) as compared to their counterparts without HAp (Figure 7a,c,e,g,i,k,m,o). Additional culture of MSCs on these biomaterials further increased the Alizarin Red S stain (Figure 7j,l,n,p). These results confirmed the observation obtained from in vitro experiments that HAp increased the mineralization of these biomaterials. Most importantly, hMSCs seeded on these biomaterials could indeed continue to differentiate into osteoblasts under the osteogenic condition in vivo which consequently promote the mineralization of these biomaterials.

## 4. Discussion

Today, bone defects have become a public health issue due to the large number of patients affected and the high risk of developing degenerative joint diseases, such as osteoarthritis. Tissue engineering has become a new therapeutic strategy to regenerate and repair bone tissue. Synthetic polymer PCL is one of the largely used biomaterial for both soft and hard tissue. In order to improve its mechanical endurance and biocompatibility for orthopedic application, PCL is often blended with either other polymers or ceramics, such as HAp [27,28]. We recently developed a two-compartmented scaffold for the osteoarticular regeneration: the first functionalized nanofibrous compartment for the subchondral bone regeneration; and a second hydrogel-based compartment mixed with human adult bone marrow-derived mesenchymal stem cells for the articular cartilage [7]. This study demonstrated that it is possible to recreate mineralization gradients physiologically present at the osteochondral level by using adult mesenchymal stem cells, an alginate hydrogel, and nanofibrous membranes of synthetic PCL polymer or collagen equipped with nanoreservoirs. We also reported that several natural or synthetic nanofibrous biomaterials, functionalized by our patented nanoreservoir technology with bioactive molecules, could be used for bone regeneration [29,30,31,32,33,34,35]. Here, we reported the characterization of four biomaterials obtained by electrohydrodynamic techniques. The objective of this study is to investigate, in combination with MSC, the potential use of these biomaterials for subchondral bone and/or cartilage regeneration.

We previously obtained, by electrospinning PCL, fibers loaded with hydroxyapatite particles which could promote the apatite formation [36]. We also decorated these fibers with biocompatible polymeric particles (PLGA) loaded with an active biomolecule (bone morphogenetic protein 2, BMP-2) to be used for bone repair [26]. Using the same fabrication procedure in this case, we obtained scaffolds consisting of hydroxyapatite loaded PCL fibers decorated with PVP particles loaded with HA. The composite scaffolds produced present the characteristic morphology that mimics the hierarchical architecture of extracellular matrix (Figure 1c), as the porous structure is needed for osteoblast migration and proliferation [37]. The presence of HAp in fibers provides a rough surface that favors the cell adhesion and proliferation [38]. Importantly, high loading efficiencies were achieved for both hydroxyapatite and sodium hyaluronate, which is in agreement with other studies [39,40,41]. It was also made sure that the sterilization procedures did not modify the nanostructure of all scaffolds produced by electrospinning.

We demonstrated that all the four tested biomaterials are biocompatible both in vitro and in vivo. AlamarBlue^®^ assay revealed that human bone marrow-derived MSCs could proliferate on these four biomaterials in both osteogenic and chondrogenic conditions. It was also suggested that differentiation of MSCs could occur in osteogenic condition, whereas the differentiation of MSCs on these biomaterials in the chondrogenic condition was not obvious (Figure 2). Under the osteogenic condition, indirect immunofluorescence showed the nuclear staining of the bone specific marker RUNX2 and the osteoblast differentiation specific marker BSPII on all the four biomaterials (Figure 3), and quantitative PCR analyses revealed a significant increase of the expression of BSPII, RUNX2, and ALP genes with biomaterials containing HAp (Figure 4 and Supplementary Figure S1). These results clearly demonstrated that MSCs could indeed differentiate into osteoblasts. Interestingly, under the chondrogenic condition, the expression of a cartilage specific marker COLII was also observed for all the four biomaterials, indicating the differentiation of MSCs into chondrocytes has taken place, as well. The different profile of metabolic activities of MSCs observed in osteogenic and chondrogenic medium maybe suggested the condition used in this study for MSC chondral differentiation was not optimal. Consequently, MSCs preferred to proliferate than to differentiate into chondrocytes in such condition. We and others have reported that hyaluronic acid could enhance chondrogenic differentiation [7,42,43]. Surprisingly, no active effect of HA was observed for the chondrogenic differentiation (Figure 3 and Figure 4). Although the electrospraying is considered as a mild technique to incorporate bioactive molecules to particles, we could not exclude the possibility for the inactivation of the HA during the process. In addition, since several functional groups are presented in sodium hyaluronate molecules and in the polymer, the inactivation of the HA activity could also due to the interaction between these chemical compounds.

Importantly, in vitro Alizarin Red S stain confirmed that the presence of HAp could not only increase the mineralization of biomaterials (Figure 5) but also promote a synergy effect, in this given osteogenic environment, for the MSCs differentiation into osteoblasts, which has also been demonstrated by immunofluorescence assay.

Notably, in vivo experiments demonstrated that these four membranes could be used as implantable biomaterials for tissue engineering because no foreign body granulomas, no scarring incidents, nor signs of rejection were observed at the implantation site upon subcutaneous implantation of these biomaterials in mice (Figure 6). In addition, peripheral vascularization was also detected within 2 weeks indicating a good integration of these biomaterials into the surrounding tissue (see Supplementary Figure S3). Most importantly, in vivo mineralization was observed in all these membranes by Alizarin Red S stain (Figure 7), which could be further strengthened by the presence of both HAp and osteoblasts differentiated from MSCs in an osteogenic environment. These results demonstrated not only a good biocompatibility and biointegration but also good osteoinduction and osteoconduction of our biomaterials for potential subchondral bone regeneration. Furthermore, no tested biomaterials were completely resorbed within two-weeks’, or even four-weeks’, implantation (see Supplementary Figure S4), suggesting a slow degradation rate of the scaffolds, which is actually required for tissue engineering. However, to determine the exact in vivo degradation time for these biomaterials, long-term implantation experiments need to be done.

## 5. Conclusions

Bioactive molecules HAp and/or HA were efficiently loaded to PCL/PVP nanofibrous biomaterials. The tested four PCL/PVP-based biomaterials could be safely used in combination with MSCs as implantable therapeutics for tissue engineering. Human bone marrow-derived MSCs could proliferate and differentiate both in vitro and in vivo on these biomaterials. The PCL-HAp could effectively increase the mineralization and induce the differentiation of MSCs into osteoblasts in an osteogenic condition. These results indicate that PCL-HAp biomaterials combined with MSCs could be a beneficial candidate for subchondral bone regeneration.

## Figures and Tables

**Table 1:** Theoretical composition of prepared scaffolds.

Scaffold	Composition (wt.%)			
	PCL	PVP	HAp	HA
PCL (S4-1)	90.79	9.21	-	-
PCL-HAp (S4-2)	56.47	5.88	37.65	
PCL-HA (S4-3)	90.58	9.19	-	0.24
PCL-HAp-HA (S4-4)	56.39	5.87	37.59	0.15

**Table 2:** List of primers used.

Gene Product	Forward Primer Sequence	Reverse Primer Sequence
ALP	CCACGTCTTCACATTTGGTG	GCAGTGAAGGGCTTCTTGTC
BSPII	GAGTGAGAGGGCAGAGGAAA	CGTGGCCTGTACTTAAAGACC
RUNX2	CCAACCCACGAATGCACTATC	TAGTGAGTGGTGGCGGACATAC
COLII	CGTCCAGATGACCTTCCTACG	TGAGCAGGGCCTTCTTGAG
Beta-actin	GATGAGATTGGCATGGCTTT	CACCTTCACCGTTCCAGTTT

## Supplementary Materials

The following are available online at https://www.mdpi.com/1996-1944/13/14/3087/s1, Figure S1 Relative gene expression of ALP, BSPII, and RUNX2 genes in hBM-MSCs cultured for 3 days in osteogenic condition and COLII gene for 3 days in chondrogenic condition. *** *p* < 0.01 as compared to PCL, Figure S2 Staining with Alkaline Phosphatase after 7 days of culture of hBM-MSCs on the two types of scaffolds (PCL and PCL-HAp) in an osteogenic medium. Bars represent 4 mm in (A,C) and 50 µm in (B,D), Figure S3 In vivo subcutaneous implantation of a sterilized scaffold for two weeks. (A,B) Macroscopic analyses showing the good integration and vascularization of the implant, (C,D) Haematoxylin-Eosin (HE) staining, yellow zone: implanted membrane; red zones: blood vessels; blue zones: immune cell aggregates; and white asterisk: fibrocytes, Figure S4 Haematoxylin-Eosin (HE) staining (a–h) and histological staining with Alizarin Red S (i–p) for PCL, PCL-HAp, PCL-HA, and PCL-HAp-HA scaffolds subcutaneously implanted for 4 weeks without cells in ICR mice. S4-1 in (a,e,i,m) for the PCL scaffold S4-2 in (b,f,j,n) for the PCL-HAp scaffold; S4-3 in (c,g,k,o) for the PCL-HA scaffold; and S4-4 in (d,h,l,p) for the PCL-HAp-HA scaffold. Black arrowheads indicate vascularization. Black squares indicate the enlarged areas.
