# Supplementary material for: Potential Implantable Nanofibrous Biomaterials Combined with Stem Cells for Subchondral Bone Regeneration
Source: Materials (Basel). 2020 Jul 10;13(14):3087. doi: 10.3390/ma13143087 (PMC7412392; doi:10.3390/ma13143087)
Supplement: Supplementary file 1 [file materials-13-03087-s001.pdf]

materials

MDPI

*Supplementary Information*

# Potential Implantable Nanofibrous Biomaterials Combined with Stem Cells for Subchondral Bone Regeneration

**Rana Smaida[1,2,†], Luc Pijnenburg [1,†], Silvia Irusta [3,4,5,†], Erico Himawan [3], Gracia Mendoza [4,5], Ezeddine Harmouch [1,2], Ysia Idoux-Gillet [1,2], Sabine Kuchler-Bopp [1,2], Nadia Benkirane-Jessel [1,2,*] and Guoqiang Hua[1,2,*]**

[1] French National Institute of Health and Medical Research (INSERM), UMR 1260, Regenerative Nanomedicine (RNM), FMTS, 11 rue Humann, 67000 Strasbourg, France; rana.smaida@etu.unistra.fr (R.S.); luc.pij@gmail.com (L.P.); ezeddine.harmouch@etu.unistra.fr (E.H.); yidouxgillet@unistra.fr (Y.I.-G.); kuchler@unistra.fr (S.K.-B.)

[2] Faculté de Chirurgie Dentaire de Strasbourg, Université de Strasbourg, 8 rue Sainte-Elisabeth, 67000 Strasbourg, France

[3] Department of Chemical Engineering. Aragon Institute of Nanoscience (INA), University of Zaragoza, Campus Río Ebro-Edificio I + D, C/ Poeta Mariano Esquillor S/N, 50018 Zaragoza, Spain; sirusta@unizar.es (S.I.); erico.himawan@gmail.com (E.H.)

[4] Aragon Health Research Institute (IIS Aragon), 50009 Zaragoza, Spain; gmendoza@iisaragon.es

[5] Networking Research Center on Bioengineering, Biomaterials and Nanomedicine, CIBER-BBN, 28029 Madrid, Spain

* Correspondence: nadia.jessel@inserm.fr (N.B.-J.); g.hua@unistra.fr (G.H.)

† These authors equally contributed to this work.

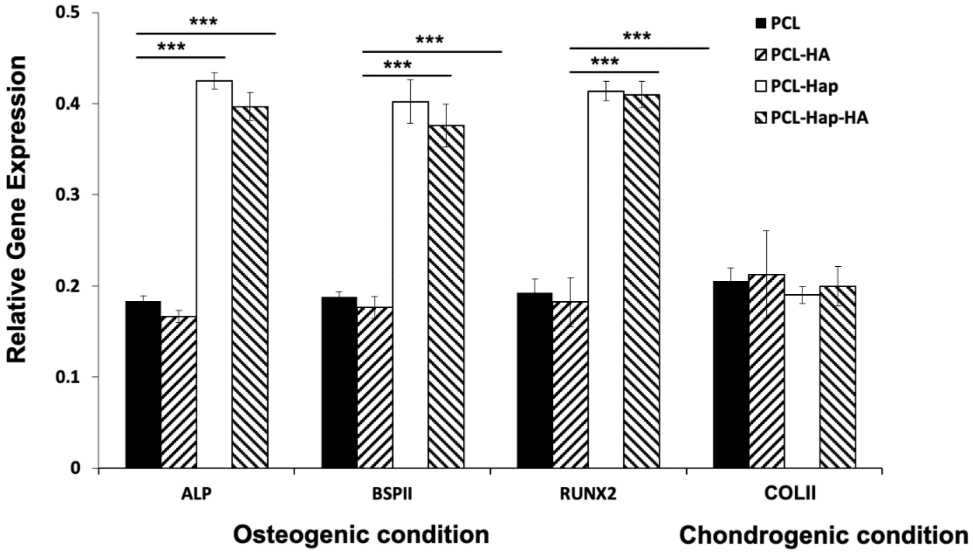


**Figure S1.** Relative gene expression of ALP, BSPII, and RUNX2 genes in hBM-MSCs cultured for 3 days in osteogenic condition and COLII gene for 3 days in chondrogenic condition. *** $p < 0.01$ as compared to PCL.

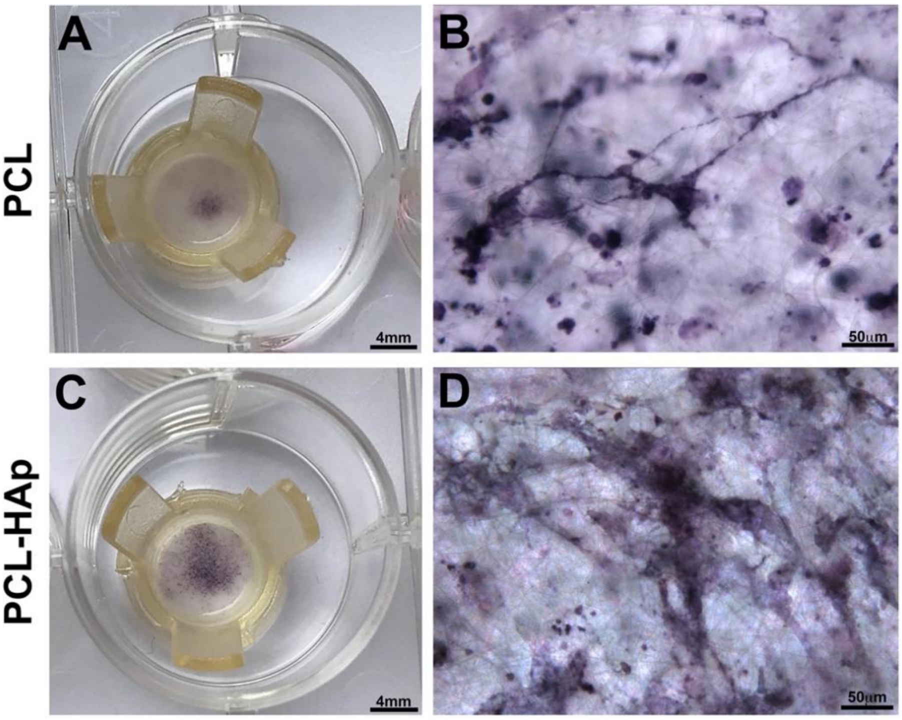


**Figure S2.** Staining with Alkaline Phosphatase after 7 days of culture of hBM-MSCs on the two types of scaffolds (PCL and PCL-HAp) in an osteogenic medium. Bars represent 4 mm in (**A**,**C**) and 50 µm in (**B**,**D**).

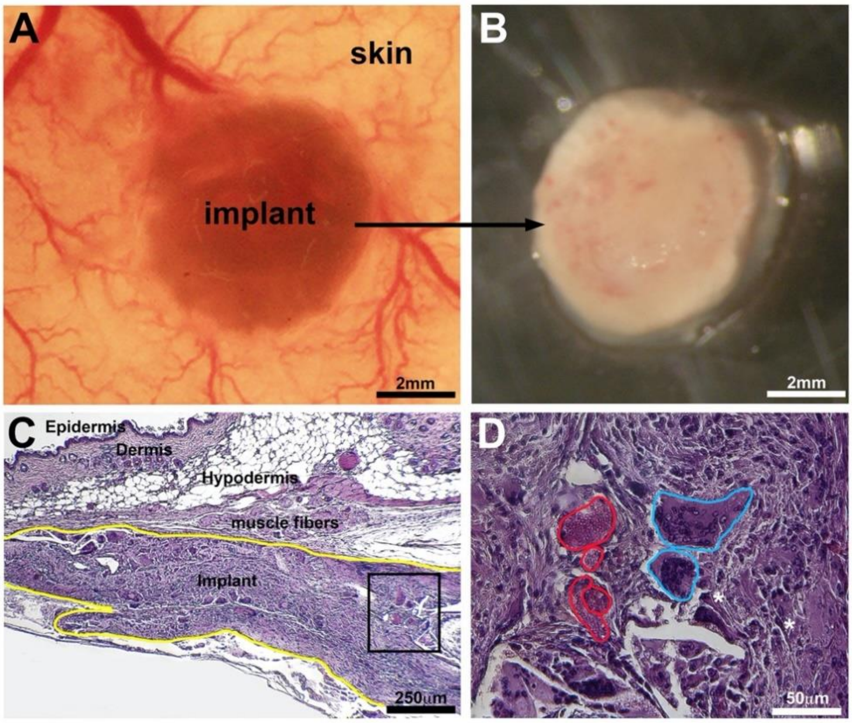


**Figure S3.** In vivo subcutaneous implantation of a sterilized scaffold for two weeks. (**A**,**B**) Macroscopic analyses showing the good integration and vascularization of the implant, (**C**,**D**) Haematoxylin-Eosin (HE) staining, yellow zone: implanted membrane; red zones: blood vessels; blue zones: immune cell aggregates; and white asterisk: fibrocytes.

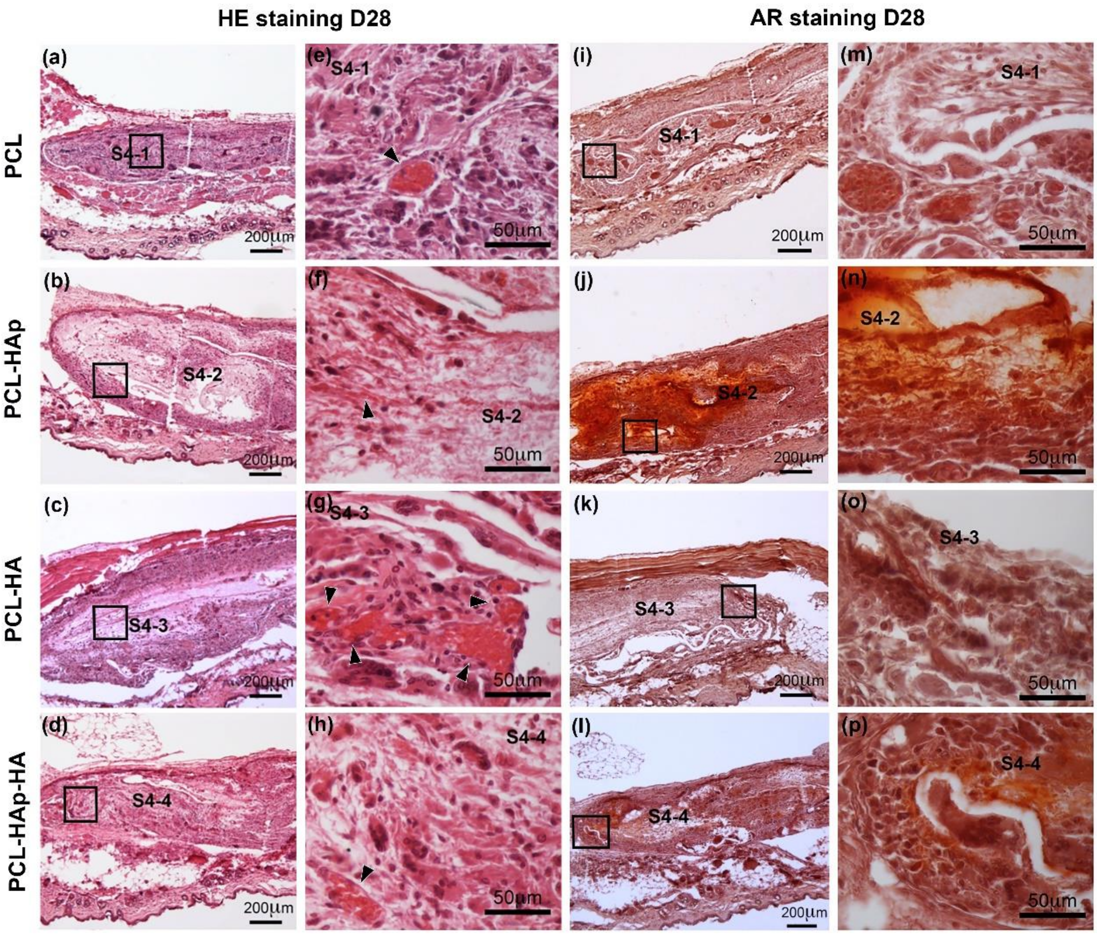


**Figure S4.** Haematoxylin-Eosin (HE) staining (**a**–**h**) and histological staining with Alizarin Red S (**i**–**p**) for PCL, PCL-HAp, PCL-HA, and PCL-HAp-HA scaffolds subcutaneously implanted for 4 weeks without cells in ICR mice. S4-1 in (**a**,**e**,**i**,**m**), for the PCL scaffold S4-2 in (**b**,**f**,**j**,**n**), for the PCL-HAp scaffold; S4-3 in (**c**,**g**,**k**,**o**) for the PCL-HA scaffold; and S4-4 in (**d**,**h**,**l**,**p**) for the PCL-HAp-HA scaffold. Black arrowheads indicate vascularization. Black squares indicate the enlarged areas.

© 2020 by the authors. Licensee MDPI, Basel, Switzerland. This article is an open access article distributed under the terms and conditions of the Creative Commons Attribution (CC BY) license (http://creativecommons.org/licenses/by/4.0/).
